# Estimating mortality of small passerine birds colliding with wind turbines

**DOI:** 10.1038/s41598-023-46909-z

**Published:** 2023-12-04

**Authors:** A. L. K. Nilsson, S. Molværsmyr, A. Breistøl, G. H. R. Systad

**Affiliations:** Norwegian Institute for Nature Research, Thormøhlensgate 55, 5006 Bergen, Norway

**Keywords:** Ecology, Climate sciences, Ecology, Environmental sciences

## Abstract

As demand for renewable energy is rising, wind power development is rapidly growing worldwide. In its wake, conflicts arise over land use changes converting pristine nature into industrial power plants and its associated adverse biodiversity effects, crowned by one of the most obvious and deadly consequences: bird collisions. Most post-construction studies report low levels of avian mortality, but the majority of these studies are conducted primarily on larger birds. However, the diversity and abundance of small passerine birds are rarely reflected in the carcass surveys, although they in numeric proportion to their abundances should be the most numerous. The assumption that surveys find all carcasses seems thus rarely fulfilled and passerine mortality is likely to be grossly underestimated. We therefore designed an experiment with dummy birds to estimate mortality of small-bodied passerines and other small-bodied birds during post-construction surveys, tested in a medium-sized wind farm in western Norway. The wind farm was surveyed weekly during the migration periods by carcass survey teams using trained dogs to find killed birds. The dogs in the carcass surveys were more successful in locating the large than the small dummy birds (60–200 g), where they found 74% of the large dummy birds. Detecting the smaller category (5–24 g) was more demanding and the dogs only found 17% of the small dummy birds. Correcting the post-construction carcass survey outcome with the results from the experiment leads to an almost fourfold increase in estimated mortality rates, largely due to the low detection rate of the smallest category. The detection rates will naturally vary between wind farms, depending on the specific habitat characteristics, the efficiency of the carcass surveys and the search intervals. Thus, implementing a simple experiment with dummy birds to future post-construction surveys will produce more accurate estimates of the wind turbine mortality rates, and thus improve our understanding of the biodiversity effects of conforming to a more sustainable future.

## Introduction

As the consequences of climate change are becoming more apparent, the global demand for renewable energy is rapidly rising^1^. Among the currently available renewable alternatives, wind power is increasingly seen as the most feasible solution at hand. Compared to hydropower with its extensive damming preventing the natural river flow severely impacting movements of aquatic organisms^2,3^, wind power has seemingly a lower overall environmental impact in the public eye. However, the development of onshore wind farms represents yet another claim on terrestrial land use and there is growing concern about the impact on terrestrial biodiversity, particularly airborne animals such as birds, bats and insects^4^. While wind farms can induce displacement due to disturbance or act as barriers to migrating birds, which might ultimately result in habitat loss and increased costs of migration, the most obvious consequence of wind power development is bird collisions^5^.

Bird collisions represent a source of direct mortality to birds; birds can collide with rotor blades, towers, the nacelle or other associated structures. Collisions can be a significant cause of mortality with potential population consequences^6^. Raptor populations are particularly susceptible, but also seabird populations have been affected by collision mortality^7–9^. Strategic planning and careful turbine placements might be one of the most important mitigation measures to reduce collision mortality. However, most studies report relatively low levels of collision mortality^5,6,10–14^, but the primary focus of studies of collision mortality, especially in Norway, has been on large birds, perhaps because they often are large-bodied and easier to find in carcass surveys^10,15,16^. The collision rates by raptors are certainly alarming in light of their comparatively low reproductive rate and small population sizes^17^. Passerine birds, on the other hand, comprise the clear majority of the birds on Earth, both in species numbers and abundances, and as such they are likely also colliding more often with wind turbines than other groups of birds^18,19^. Small passerines as well as other small-bodied birds are however often seriously underrepresented in the collision statistics, as pointed out by Rydell et al.^4^ and Grünkorn et al.^20^. However, Ericksen et al.^21^ shed light on wind energy mortality in small passerine birds. Since passerines in general are smaller, their carcasses are more difficult to find underneath the turbines, and they also decompose faster than larger birds^5,22^. Thus, there remain considerable uncertainty in whether all carcasses are in fact found during the carcass surveys in post-construction studies^5^.

Carcass surveys are surprisingly often made by human observers^12,18,23^ instead of using trained dogs which are proven to be distinctly more efficient in locating carcasses^14,24–26^. Trained dogs efficiently search an area faster than humans relying only on visual cues, thus enabling searches to be both time- and cost-effective. Therefore, surveys can therefore be conducted at shorter intervals. Irrespective of survey method, most studies assume all carcasses are found, which might not be true, especially not for surveys conducted by humans or where scavengers are efficient in removing carcasses. A few studies estimate the rate of carcass removal by scavengers^14,27,28^, but few studies estimate the search efficiency of the carcass surveys. Estimating search efficiency is imperative, because it allows estimation of the true wind turbine mortality and therefore a more accurate assessment of the biodiversity effects of wind power development^23^. Such an approach is thus not about controlling the search parties’ efficiency, irrespective of whether they are composed by humans or dogs. Estimating search efficiency in order to assess turbine mortality is particularly important at wind power developments situated at migratory pathways with millions of passing small-bodied migrants.

Most studies find few^6,14,29^ or no dead small-bodied birds but see^21,23^. Because we had access to a post-construction survey scheme with intense searches by skilled dog equipages able to find the bodies of the small-bodied migrants colliding with the turbines in the wind farm, we were able to design a simple experiment to estimate small passerine search efficiency, which easily can be implemented in any post-construction study. We conducted the experiment at an average-sized wind power development in a coastal mountainous region in western Norway where there is intense seasonal migration of primarily passerine birds^30^.

## Results

Dummy birds are defined as dead, small passerine, and in a few cases small non-passerine birds that were flown out to under the turbines by a drone and recovered by the carcass survey teams in 2021. Small dummy birds comprised 5–24 g birds, whereas large dummy birds comprised 60–200 g birds. Among the 47 flown-out dummy birds, only a total of 21 (45%), were found by the carcass surveys. However, the search parties found as many as 74% of the larger dummy birds (17 of 23), but only 17% (four of 24) of the smallest dummy birds (Χ^2^ = 4.9, df = 1, P = 0.027).

In the post-construction survey the dummy experiment was conducted under, 54 and 51 turbine-killed small-bodied birds were found under the turbines in 2021 and 2022, respectively (Table 1). In 2021, the killed birds of the same size as the large dummy birds were 22, and 29 birds of the same size as the small dummy birds, in addition to three larger birds. In 2022, 11 were of the same size as the large dummy birds, 29 of the same size as the small dummy birds, and 11 larger birds. The majority of carcasses, 87% (91 of 105), were small passerine birds. Five of the carcasses were partly decomposed or only parts of them were found. Four carcasses of Willow Ptarmigan *Lagopus lagopus* were found intact, but at such a distance from the turbine base as to indicate a collision with the tower. At some turbines no killed birds were detected, whereas other turbines killed as many as four birds per migratory period. The maximum number of killed birds by one single turbine was six, two in spring and four in autumn.

**Table 1:** The number and species of the turbine-killed birds found under the turbines at Guleslettene Wind Farm during spring migration (March 15th–May 20th) and autumn migration 2021 (July 15th–October 31st), respectively.

Species	2021		2022	
	Spring	Autumn	Spring	Autumn
Eurasian Teal (*Anas crecca*)				1
Willow Ptarmigan (*Lagopus lagopus*)		1	3	1
Grouse sp. (*Lagopus* sp.)			1	1
Black Grouse (*Lyrurus tetrix*)			1	
Great Cormorant (*Phalacrocorax carbo*)				1
White-tailed Eagle (*Haliaeetus albicilla*)				1
European Golden Plover (*Pluvialis apricaria*) NT**		2		2
Jack Snipe (*Lymnocryptes minimus*)**		3		
Common Snipe (*Gallinago gallinago*)**	1	1	1	
Great Black-backed Gull (*Larus marinus*)	2			
Common Wood Pigeon (*Columba palumbus*)			1	
Meadow Pipit (*Anthus pratensis*)*		6		2
White Wagtail (*Motacilla alba*)*			2	
Eurasian Wren (*Troglodytes troglodytes*)*			2	
European Robin (*Erithacus rubecula*)*	2	1	1	
Redwing (*Turdus iliacus*)**		12		3
Song Thrush (*Turdus philomelos*)**			1	
Fieldfare (*Turdus pilaris*)**				1
Common Blackbird (*Turdus merula*)**	2		1	
Ring Ouzel (*Turdus torquatus*)**			1	
Willow Warbler (*Phylloscopus trochilus*)*	2	4		2
Common Chiffchaff (*Phylloscopus collybita*)*		1		
Goldcrest (*Regulus regulus*)*		9	1	10
Spotted Flycatcher (*Muscicapa striata*)*				1
European Pied Flycatcher (*Ficedula hypoleuca*)*			1	
Eurasian Treecreeper (*Certhia familiaris*)*				1
Common Starling (*Sturnus vulgaris*) NT**	1			
Eurasian Chaffinch (*Fringilla coelebs*)*	1		1	
Brambling (*Fringilla montifringilla*)*		2	1	
Red Crossbill (*Loxia curvirostra*)*				1
Passerine sp. (*Passeriformes* sp.)		1	1	3
**Sum**	**11**	**43**	**20**	

Red List status is marked in bold, based on the Norwegian Red List of threatened species^42^.

*Indicates killed birds of the size of the small dummy birds.

**Indicates killed birds of the size of the large dummy birds.

By applying the results from the dummy study to the post-construction study results in 2021 and 2022, we estimate the accurate turbine mortality for the large dummy-sized birds where there was found 22 and 11, respectively, and where the detection rate was 0.74, as 33/0.74 = 44.6. The post-construction study found 29 small dummy-sized birds in each of the study years, where the detection rate was 0.17, and the real turbine mortality for this category was thus estimated to 58/0.17 = 341.2. Including the 14 larger birds found in the carcass surveys, this amounts to 400 dead birds in total during the two years of annual migration, including two spring migrations and two autumn migrations. Thus, the turbine mortality of small-bodied birds (large and small dummy-sized birds) from the carcass surveys alone were grossly underestimated, since based on the dummy results, the real turbine mortality was almost four times as high. The real turbine mortality equals 0.77 dead birds per turbine per migration month, or 0.62 dead birds per produced GWh.

Among the dummy birds found by the carcass surveys, most were found within the first week after placement (14 of 21; 67%). There was no statistically significant difference in the number of days until dummy birds were found between large and small dummy birds (df = 3.2, t = 1.9, P = 0.154), although the average for large and small dummy birds were 5.2 days (SD = 4.7) and 15.7 days (SD = 11.2), respectively.

Among the 26 dummy birds not found by the carcass surveys, seven positions could not be relocated in a manual search at the end of the autumn season. This might be due to poor drone pictures, snow cover having altered the appearance of the position, scavenger removal, or few landmarks around the dummy birds on the drone picture. Among the 19 dummy birds that were relocated on this manual search, 11 were found on the same position or in the immediate surroundings (one was swept off 1–2 m by a small brook). 10 of the 11 relocated dummy birds in the same position or in the immediate surroundings were small dummy birds. The only remaining large dummy bird, flown out in October was not found until the next summer after the dummy experiment was concluded, and not included in the present calculations.

**Figure 1:**
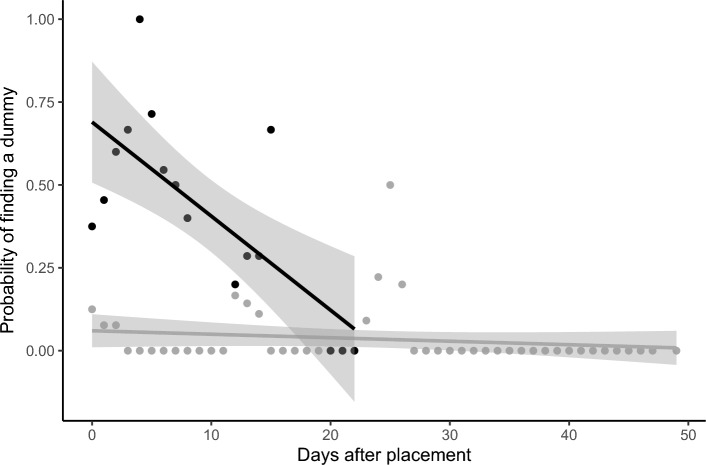
Observed probability of locating a dummy bird on the carcass surveys N days after they were placed under the turbines at Guleslettene Wind Farm. The datapoints are aggregated for the whole season. Black = large dummy birds, grey = small dummy birds.

**Figure 2:**
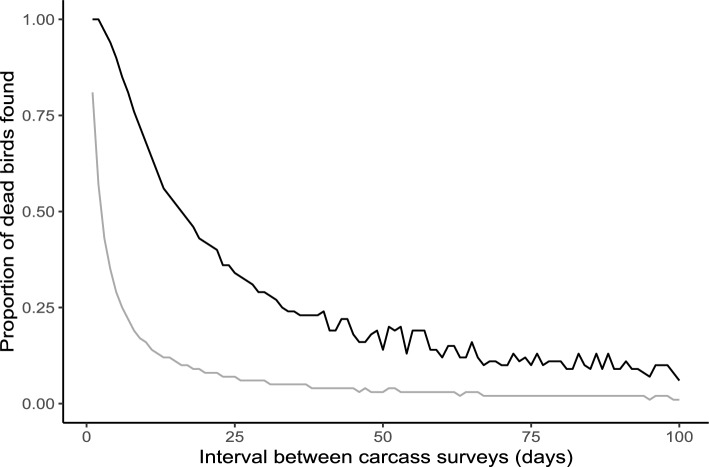
The proportion of all dead birds found, based on data from Guleslettene Wind Farm and a simulation with different search intervals. The proportion of birds found drops as time progress, as the likelihood of finding each individual approach zero. Black = large dummy birds, grey = small dummy birds.

We also estimated the detection rate of dummy birds over time (large dummy birds: df = 14, R^2^ = 0.53, a = 0.72, b =  − 0.03, t =  − 4.0, P = 0.001; small dummy birds: df = 47, R^2^ = 0.03, a = 0.06, b =  − 0.001, t =  − 1.2, P = 0.242; Fig. 1) and simulated how rapidly the proportion of dead birds found falls with longer intervals between carcass surveys (Fig. 2). For the small dummy-sized birds the proportion falls very rapidly, and high search frequencies are required to locate the carcasses before they decompose or are removed by scavengers (Fig. 2). For the large dummy-sized birds the proportion does not fall as rapidly with longer search intervals, although it has declined to 25% detected at 1 month search intervals. For small dummy-sized birds, the detected proportion was even lower (Fig. 2). Thus, to locate passerine migrants, it is necessary with short intervals between the carcass surveys, such that each turbine is searched with high frequency.

## Discussion

Here, we demonstrate how a simple addition to a post-construction survey design can increase the accuracy of estimates of biodiversity impacts of wind power developments. There was a clear difference in how many of the large versus the small dummy-sized birds were found by the carcass surveys; search efficiency was considerably higher for the large dummy birds than for the small ones. Based on search efficiency, we could calculate more accurate estimates of the biodiversity impacts, here measured as turbine mortality. Despite very skilled carcass survey teams, real turbine mortality for small-bodied birds amounted to almost four times as high as what it would have been if the biodiversity impact had solely been based on what was found by the carcass surveys. The lower detection of the small dummy birds contributed the most to the considerable increase in the estimates; even the large dummy birds which consisted of small birds the size of Common Starlings *Sturnus vulgaris* and thrushes *Turdus* sp. were easily detected by the carcass surveys in the rough terrain. Thus, all birds larger than the dummy birds were most likely found. However, the detection probabilities found here are site-specific, thus they only apply to Guleslettene Wind Farm, and also the first years after the wind farm was built. The high frequency of the carcass surveys contributed to the surprisingly high detection rate of all small-bodied birds in this post-construction study.

Increasing the accuracy of biodiversity impact estimates by conducting a simple experiment during the post-construction study is particularly relevant at wind power developments in habitats that are challenging to conduct carcass surveys in. Few Scandinavian wind power developments are found in habitats so highly suitable for carcass surveys such as the habitat for instance Aschwanden et al.^23^ conducted their detailed study in, with closely mowed grass lands ideal for carcass survey experiments. Many Norwegian wind power developments and indeed many wind power sites around the world, are situated at exposed mountain plateaus with bare mountain and rocks, with cliffs and crevices, and sparse vegetation, which are extremely challenging to conduct carcass surveys in, even in good weather. Such terrain likely also influences the detection rates because carcasses might fall down into cracks and crevices which might be almost impossible to access, thus emphasizing the difficulty of not only performing carcass surveys but also the likelihood of finding and locating carcasses. This further stress the need for trained dogs in the carcass surveys teams.

In challenging survey conditions, it is inherently difficult to conduct carcass surveys especially after small-bodied birds. Most post-construction surveys find few, if any, small-bodied birds^14,20,22,28^. This makes post-construction studies of wind power developments at migratory passage sites particularly awkward, because the bulk of migrants are small-bodied passerine birds^31^ (but see^32^). At the post-construction survey at Guleslettene Wind Farm, despite the particularly difficult terrain and exposed survey conditions, the majority of carcasses were in fact small passerine birds, and as was evident from this, the search efficiency was therefore surprisingly high. Although most carcasses were of migratory birds, some of the carcasses were likely locally breeding birds or their fledged offspring. The simplest remedy for amending the exclusion of passerine birds is, as mentioned before, the use of trained dogs in carcass surveys, but, as is also evident from this study, it is also important with frequent surveys. Carcasses of small-bodied birds have a high detection probability the first days after they are killed but this declines rapidly over time. This is likely due to a combination of decomposition of the carcasses and scavengers removing carcasses. As small carcasses decompose rapidly, the scent traces that could be picked up by the dogs in the carcass surveys, especially in wet weather conditions, are reduced. Thus, anything less than carcass surveys every second week cannot be expected to truly measure turbine mortality of small-bodied birds. The rates of decomposition and scavenger removal are highly relevant for estimating turbine mortality, but estimates of rates are necessarily site-specific, meaning that the rapid decline in detection probability found here is not directly transferable to other wind parks.

There was a distinct difference in detection probability between large and small dummy birds. The dogs had seemingly little difficulty in locating most of the large dummy birds, while the small dummy birds turned out to be more challenging to detect. However, enough of the small dummy birds were located to enable a separate estimation of search efficiency and thus a more accurate estimation of turbine mortality rates during migration. The overall search efficiency of this study (45%, including all dummy birds) was actually larger than the overall search efficiency of other studies cf 38.7%^32^. Aschwanden et al.^23^ found that small birds equal in size to our small dummy birds had a detection probability of less than 50% when the vegetation height was more than 10 cm, whereas medium-sized birds equaling our large dummy birds just as in our study had a higher detection probability. An American study^33^ estimated search efficiency of the smallest birds in their study, which was comparable to our large dummy birds, to 42–55%. Neither of the comparable studies used trained dogs.

Comparisons of mortality rates between wind power developments are not straightforward, because a multitude of factors can affect the estimates. Some environments have higher bird diversity and abundances, such as wetlands and coastal regions, and are therefore also associated with intrinsically higher mortality rates^34^. Some habitats are also easier to survey and conduct experiments in than others, where grasslands are considerably less challenging than high mountainous, rocky terrain with outcrops and crevices^23,26,35,36^. Also, post-construction surveys are currently not standardized, despite the obvious need for repeatable and adequate monitoring methods^5^; there is therefore a diversity of approaches used that render comparisons of turbine mortality extremely complex. The length of the post-construction survey and its design might strongly differ, depending on conditions set by authorities or the developer’s own ethical standards, as well as the frequency and extent of carcass surveys, whether scavenger activity has been controlled for or not, whether carcass surveys are conducted by humans or trained dogs, etc.^14,18,23,34^. For instance, the post-construction survey at Guleslettene Wind Farm only included the migration periods, whereas other surveys range from comprising the full annual cycle^12,15,33,37^ to much shorter periods comprising only a few weeks^38,39^. Also, the fact that most carcass surveys fail to find the small passerines and other small-bodied birds that were the main target of the present study, and, moreover, fail to correct for search efficiency in impact measures, implies that direct comparisons are barely possible. While Rydell et al.^34^ assumed that most wind turbines kill between five and 10 birds annually, they also stated that most turbines kill very few birds, whereas a few, often ill-placed turbines kill considerably more. To mitigate biodiversity impacts, it is particularly important to detect those ill-placed turbines. Despite the listed shortcomings, an attempt to compare our more accurate estimate of turbine mortality with these rough estimates likely places Guleslettene Wind Farm below what would have been expected, despite its location at a migratory passage site and the presence of ridges providing thermals for raptors and other large soaring birds.

Implementing the study design presented here in more onshore post-construction studies will increase the present status of knowledge and moreover enable comparisons between different projects. This will ultimately lead to even better siting of onshore wind power projects in the future as society continue to work towards a more sustainable future.

## Methods

**Figure 3:**
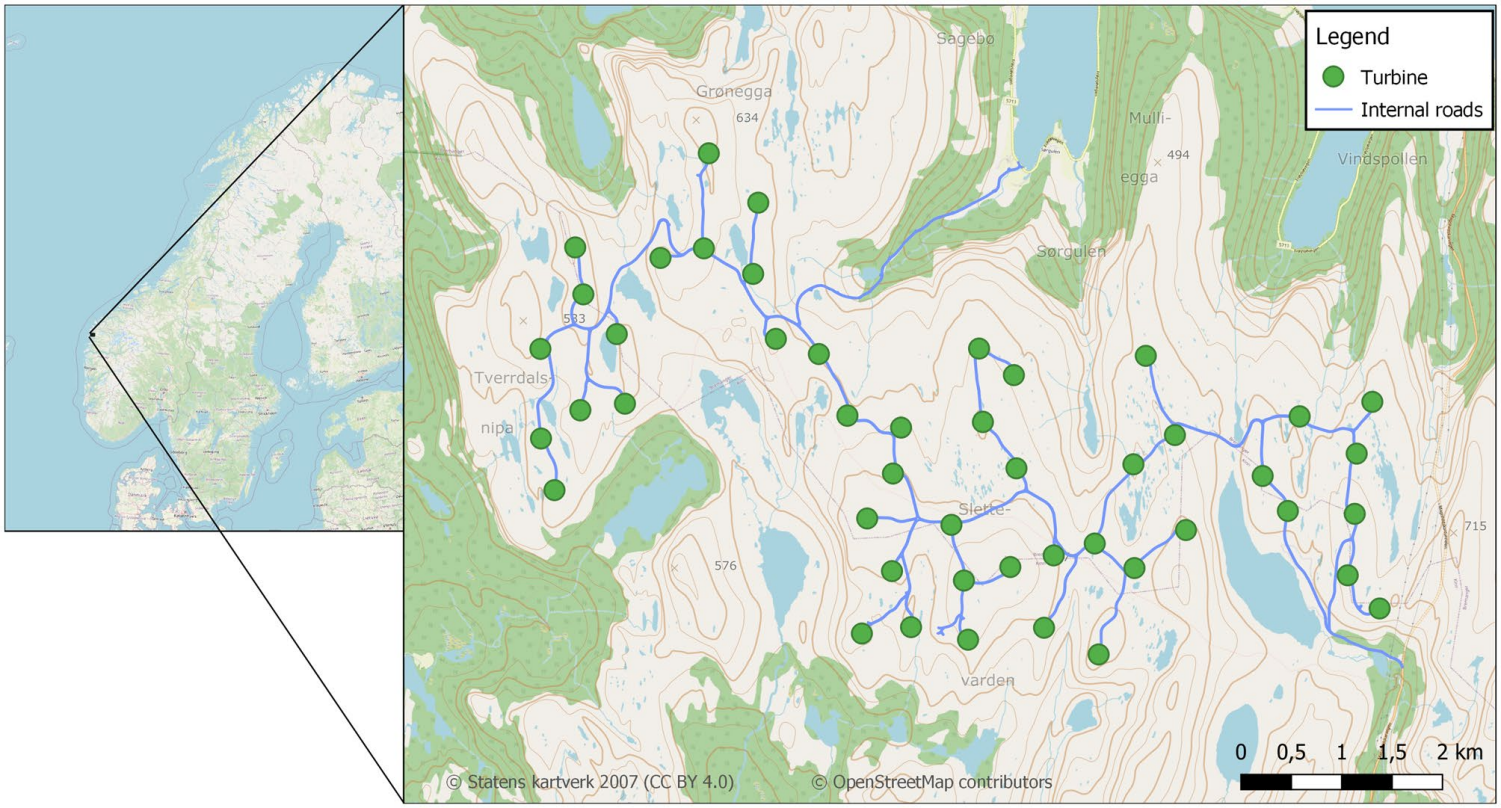
Map over the Wind farm at Guleslettene, in western Norway.

Guleslettene is a high mountain plateau close to the coast in the north of Vestland county in Norway (Fig. 3). It is characterized by bare mountain and rocks, and low vegetation of lichens, mosses and grasses. Heather (primarily European blueberry *Vaccinium myrtillus* and crowberry *Empetrum nigrum*) occurs in lower or more sheltered parts of the mountain plateau. The wind farm at Guleslettene is spread across almost the entire plateau and consists of 47 wind turbines with an annual estimated total production of 700GWh. The turbine bases rise 90m, and the rotor blade radius is 68m. The post-construction study at Guleslettene was conducted during 2021 and 2022^40^.

We carried out a dummy experiment to estimate the mortality of small-bodied passerines and other small-bodied birds during the first year of the post-construction survey. Carcass surveys for the dummy experiment was carried out at the same time as the carcass surveys for the first year of the post-construction study (2021). The post-construction study was a requirement for the wind development concession at Guleslettene. The carcass surveys were continued during the second year of the post-construction study in 2022. The carcass surveys were conducted weekly under all the turbines with the aid of trained dogs during spring (March 15th–May 15th) and autumn migration (July 15th–31st October). The carcass survey teams used one dog in each search around a turbine, although some carcass survey teams had access to more than one dog. A 100-m radius around each turbine was surveyed^41^, where half of the wind farm was covered during day 1 and the rest during day 2. In some instances, surveys were also conducted on day 3. Because of the location of the wind farm at a high, exposed mountain plateau, days with blizzards, heavy rain or storms occasionally prevented surveys to be completed as planned or had to be aborted after initiation. Each turbine was surveyed on average every 9 days (8.8 ± 3.4 days). There were 906 individual carcass surveys in 2021, measured as individual turbine searches, conducted for 65 days during 25 weeks. Each turbine survey lasted on average 20.3 min (SD = 8.2), but several of the turbines had so steep surroundings as to render it unsafe to search in the entire 100-m radius.

To avoid that the dogs would follow our tracks in the snow in early spring and any odor traces in the snow as well as on the bare ground as the season progressed, we always flew out all dummy birds using a drone. To avoid confusion with real collision fatalities, as the dummy experiment was carried out simultaneously with the post-construction carcass surveys, a thin white rope was tied to the tibia of all dummy birds. The loop of the thin rope was also used as an attachment point to the drone. All dummy birds were placed within the 100 m radius from the turbine base the dogs were searching. Most dummy birds were birds previously found by the dogs at the wind farm. In the cases where there were not enough birds available some birds were acquired from Flesland airport in Bergen or birds that had hit the windows of one of the researchers during the spring (two Eurasian Bullfinches *Pyrrhula pyrrhula*). All dummy birds were kept in a freezer before and between placement under the turbines. Some of the dummy birds were in good condition after use (meaning they were found shortly after placement) and were reused in the experiment.

In total 47 dummy birds were placed around the turbines, whereof a total of 14 were flown out successively during spring and a total of 33 likewise during the autumn (Supplementary Data 1). All dummy birds were frozen immediately after collection, both before experiments and after retrieval by the carcass survey teams. To avoid attracting raptors and other avian scavengers such as Hooded Crows *Corvus cornix* and Northern Ravens *Corvus corax* which might increase their collision rates, we only used small-bodied dummy birds. The dummy bird species were Eurasian Chaffinch *Fringilla coelebs*, Eurasian Bullfinch *Pyrrhula pyrrhula*, Fieldfare *Turdus pilaris*, European Golden Plover *Pluvialis apricaria*, Meadow Pipit *Anthus pratensis*, Willow Warbler *Phylloscupus trochilus*, Barn Swallow *Hirundo rustica*, Song thrush *Turdus philomelos*, European robin *Erithacus rubecula*, Redwing *Turdus iliacus*, Common Starling *Sturnus vulgaris and* Common Blackbird *Turdus merula*. Because of the obvious size differences, we also divided the dummy bird species into small (Eurasian Chaffinch, Eurasian Bullfinch, Meadow Pipit, Willow Warbler, Barn Swallow, and European Robin; 5–24 g) and large (Fieldfare, European Ggolden Plover, Song Thrush, Redwing, Common Starling, and Common Blackbird; 60–200 g) dummy birds for the analyses. At the close of the autumn season, we set out to locate the 26 dummy birds not found by the carcass surveys using known GPS positions and drone pictures of the original locations.

### Statistical analyses

The proportion of detected dummy birds was defined as the number of dummy birds found by the carcass survey teams divided by the total number of flown-out dummy birds of each size category. To test whether there was a difference in detection rate between large and small dummy birds, we used a Chi-square-test. We tested whether there was a difference in the number of days required to retrieve a large versus a small dummy bird with a T-test.

To find the proportion of detected birds, the time of placement and time of retrieval for each dummy bird were combined with the search occurrences for each individual turbine. For each search, we then calculated the number of days each dummy bird had been present. For each day after placement from 0 to 49 we then calculated, to avoid too many missing values, the rolling average (± 1 day) proportion of dummy birds found. This also meant that a dummy bird that was never found would count in multiple searches. To these data there were fitted linear regression models, one for large dummy birds, and one for small dummy birds.

Based on these linear regression models a simulation was performed to visualize what a real-life scenario would look like, and which proportion of the killed birds would be found with different search intervals in the carcass surveys. As actual bird deaths are unlikely to be a uniform distribution throughout the year, the turbine killed birds found at Guleslettene was used to create an estimate of how many birds was killed in the windfarm for each day. This estimate was created by taking the average of number of birds found by the real carcass surveys from 10 days before to 5 days after each date. If there were no birds found in that period (either because of no searches or no findings) the kill rate was set to 1 bird per day, both to ensure a minimum threshold for daily mortality estimates and to make the simulation less likely to miss bursts of dead birds at long search intervals. The estimated death rate per day varied between 1 and 5, and could be a decimal number. Thereafter, two basic population models of killed birds were created, one for large and one for small birds. For each population model, for each day the number of killed birds from the estimated death rates was added to the population. For simplicity we assumed a similar death rate for large and small birds. Based on this, we simulated the proportion of killed birds detected, at different search intervals, using the linear regression models of detection probability over time created before (see Supplementary Fig. S1 for an example). This included repeated searches for each dead bird, meaning that a bird not detected on the first search after its death, would still be in the population for the next search.

### Supplementary Information

Supplementary Information 1.

Supplementary Information 2.

Supplementary Information 3.

Supplementary Information 4.

Supplementary Figure S1.

## Data Availability

Data are available in the Supplementary Data file, but also upon request from the authors (anna.nilsson@nina.no).
