# Supplementary material for: Estimating mortality of small passerine birds colliding with wind turbines
Source: Sci Rep. 2023 Dec 4;13:21365. doi: 10.1038/s41598-023-46909-z (PMC10695956; doi:10.1038/s41598-023-46909-z)
Supplement: Supplementary file 5 — Supplementary Figure S1. [file 41598_2023_46909_MOESM5_ESM.docx]

**Supplementary Figure S1**


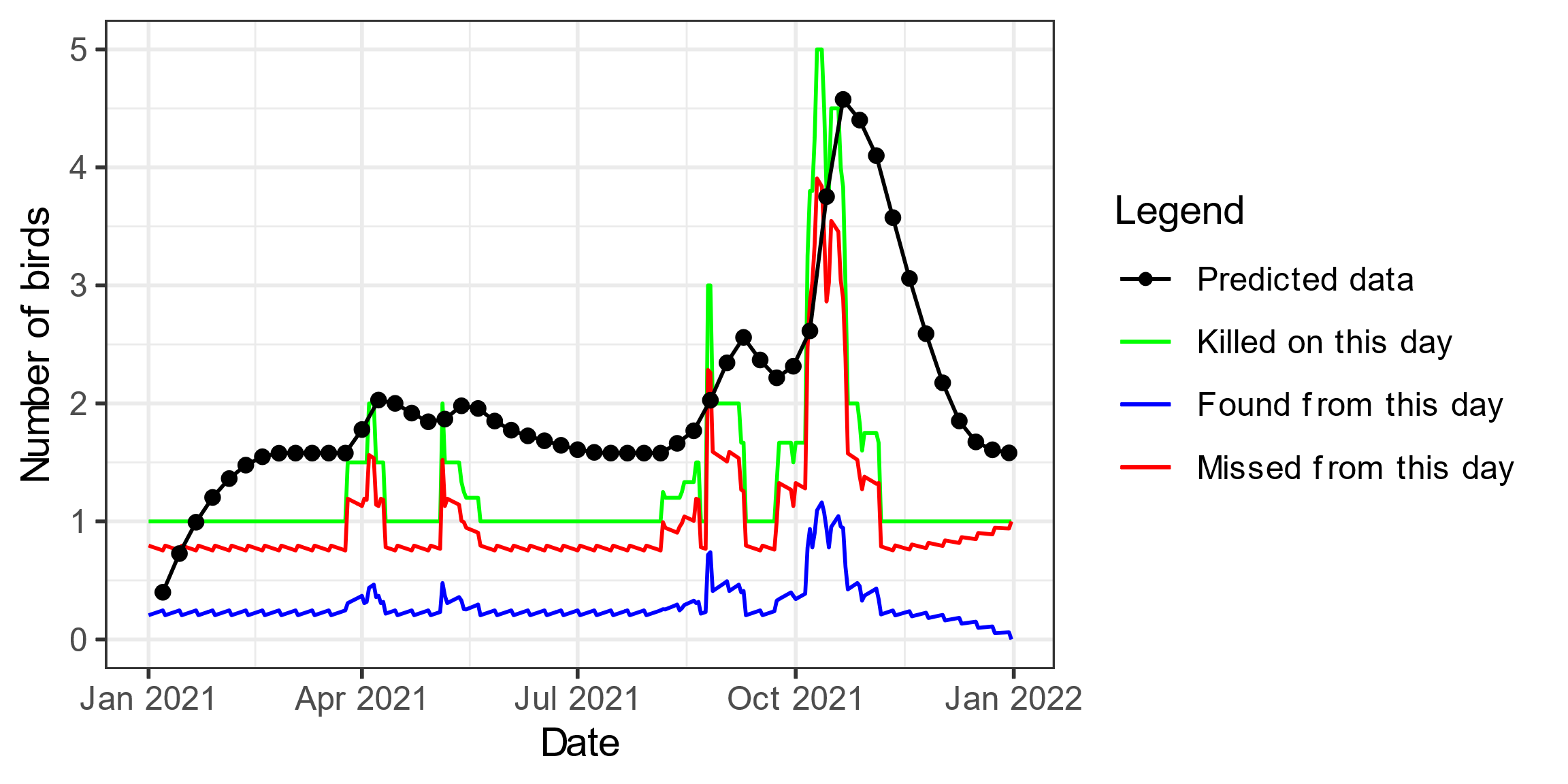


Supplementary Figure S1. The simulation of carcass surveys on small-sized dummy birds with 7 days search intervals, as an example of the processes behind Figure 2. In total 476 birds were killed in this iteration of the simulation, 104 of them were predicted to be found (22 %). Each of the colored lines represent a group . Green is the number of birds killed on each day, calculated from the real wind turbine collisions from the carcass survey during the post-construction study at Guleslettene in 2021, but with 1 bird per day as a background rate. The green line remained constant between different search intervals. The red and blue line are mirror images of each other and always add up to the green line. These shows how many of the birds killed on a given day was expected to be found by the dog searches. These lines will vary based on which search interval was simulated. The black line represents the number of birds predicted to be found by each individual carcass survey. This process was repeated for each search interval.
